# Why are some people reluctant to be vaccinated for COVID-19? A cross-sectional survey among U.S. Adults in May-June 2020

**DOI:** 10.1016/j.pmedr.2021.101494

**Published:** 2021-07-14

**Authors:** Jennifer D. Allen, Wenhui Feng, Laura Corlin, Thalia Porteny, Andrea Acevedo, Deborah Schildkraut, Erin King, Keren Ladin, Qiang Fu, Thomas J. Stopka

**Affiliations:** a Department of Community Health, Tufts University, 574 Boston Ave, Medford, MA 02155, USA; b Department of Public Health and Community Medicine, Tufts University School of Medicine, Boston, MA, USA; c Department of Civil and Environmental Engineering, Tufts University School of Engineering, 200 College Ave, Medford, MA 02155, USA; d Department of Occupational Therapy, Tufts University, 574 Boston Ave, Medford, MA 02155, USA; e Department of Political Science, Tufts University, Packard Hall, Medford, MA 02155, USA; f Department of Clinical Science, Cummings School of Veterinary Medicine, 200 Westboro Road, North Grafton, MA 01536, USA; g Tufts Clinical and Translational Science Institute, 35 Kneeland Street, Boston, MA 02111, USA

**Keywords:** COVID-19, Coronavirus, Vaccine, Attitudes, Health disparities, United States

## Abstract

Understanding reasons for COVID-19 vaccine hesitancy is necessary to ensure maximum uptake, needed for herd immunity. We conducted a cross-sectional online survey between May 29-June 20, 2020 among a national sample of U.S. adults ages 18 years and over to assess cognitive, attitudinal and normative beliefs associated with not intending to get a COVID-19 vaccine. Of 1219 respondents, 17.7% said that they would not get a vaccine and 24.2% were unsure. In multivariable analyses controlled for gender, age, income, education, religious affiliation, health insurance coverage, and political party affiliation, those who reported that they were unwilling be vaccinated (versus those who were willing) were less likely to agree that vaccines are safe/effective (Relative Risk Ratio (RRR): 0.45, 95% confidence interval (CI): 0.31, 0.66), that everyone has a responsibility to be vaccinated (RRR: 0.39, 95% CI: 0.30, 0.52), that public authorities should be able to mandate vaccination (RRR: 0.75, 95% CI: 0.58, 0.98), and more likely to believe that if everyone else were vaccinated they would not need a vaccine (RRR: 1.36, 95% CI: 1.04, 1.78). Our results suggest that health messages should emphasize the safety and efficacy of vaccines, as well as the fact that vaccinating oneself is important, even if the level of uptake in the community is high.

## Highlights

• Nearly one-in-five U.S. adults would not get a COVID-19 vaccine.
• Cognitive, attitudinal, and normative factors are associated with COVID-19 vaccine intentions.
• Campaigns should emphasize safety/efficacy, promote trust in authorities, encourage altruism.

## 1 Introduction

Prophylactic vaccines for SARS-CoV-2 are crucial to controlling the coronavirus disease 2019 (COVID-19) pandemic. However, vaccine uptake has been suboptimal in the United States (U.S.) and has slowed in recent months. (Centers for Disease Control and Prevention, 2020) While the exact percentage of the population that must be vaccinated in order to reach herd immunity is debated (Kadkhoda, 2021, Aschwanden, 2020) and varies based on population characteristics, (Anderson et al., 2020, Fontanet and Cauchemez, 2020) some have estimated that it may require as much as 50–82% of population to be vaccinated in order to prevent ongoing community transmission. (Omer et al., 2020, Iboi et al., 2020, Randolph and Barreiro, 2020) By late May 2021, nearly half of U.S. adults have had at least one vaccine dose. (Centers for Disease Control and Prevention, 2020) However, among those not yet vaccinated, recent polls suggest that 13–15% of the population do not intend to be vaccinated, a rate that has remained relatively unchanged over recent months. (Bureau, 2021) In comparison, a systematic review of surveys conducted between June-September 2020 among 33 countries found that the U.S. had among the lowest COVID-19 vaccine acceptance levels (56.9%). (Sallam, 2021) Understanding the perspectives and concerns of those who are hesitant to be vaccinated is essential to maximizing the full benefit of COVID-19 vaccines on population health in the U.S., as well as for achieving the goal of global vaccination.

Resistance to (non-COVID-19) vaccines or “vaccine hesitancy” (i.e., delay in acceptance or refusal of available vaccines) has been on the rise in the U.S. and elsewhere, a trend the World Health Organization considers a major threat to global health. (Jarrett et al., 2015) The COVID-19 pandemic has created the perfect storm to fuel vaccine fears in the U.S.: it is a novel virus with unknown origins, mistrust of government is high (Pew Research Center, 2021) and there is intense political polarization across the country. (Iyengar and Krupenkin, 2018, Pew Research Center, 2017) Moreover, vaccine development efforts have proceeded at an unprecedented pace, creating the unfounded perception that shortcuts may have been taken or that there is a lack of transparency in the testing process. (Torreele, 2020, Kaiser Family Foundation, 2020) Initial roll-out of the three currently-available COVID-19 vaccines in the U.S. has met with numerous challenges, including lower-than-expected uptake among medical professionals, (American Nurses Association, 2021) misinformation circulating on social media, (Wilson and Wiysonge, 2020, Islam et al., 2021) and a recent short-term and now lifted pause on the Johnson and Johnson vaccine due to safety concerns. (Remmel, 2021)

To inform the current and potential future 'booster' COVID-19 vaccination efforts, we conducted a national survey designed to be representative of the adult U.S. population that assessed factors associated with hesitancy to be vaccinated. We hypothesized that individuals who were hesitant about vaccination would be more likely to report concerns about vaccine safety and efficacy, to believe that it is not an individual or societal responsibility to be vaccinated, and would express lower levels of trust in public officials who recommend or mandate vaccination. We also hypothesized that these relationships would be modified by political party affiliation, since there is major political polarization in the U.S. and efforts to address the pandemic have been highly politicized, with Democrats generally being more amenable to mitigation efforts, such as masking, compared with Republicans. (Kaiser Family Foundation, 2020)

## 2 Methods

### 2.1 Data

Our cross-sectional analysis used data from the Equity in Health, Wealth, and Civic Engagement Survey designed by Tufts University and administered between May 29th to June 10th, 2020. This survey was fielded in English and Spanish by Ipsos, a social science company. Ipsos uses a web-enabled Panel (KnowledgePanel®), and is the largest, online, probability-based panel representative of the U.S. population. The Panel was first developed in 1999 by Knowledge Networks®, an Ipsos company, using random digit dialing. In April 2009, in response to the growing number of cellphones only households, Ipsos migrated to address-based sampling. An advantage of the Ipsos Panel is that members were randomly selected to represent the U.S. population with a measurable level of accuracy, a feature that is not obtainable from non-probability or opt-in online panels. (MacInnis et al., 2018, Yeager et al., 2011, Quirks.com, 2021). After initially accepting the invitation to join the Panel, respondents were asked to complete a short demographic survey (Core Profile Survey). With privacy and confidentiality protections, respondents then became active Panel members. Estimated annual attrition of Panel members is about 18%. (KnowledgePanel. Ipsos. Accessed July 6, 2021)

We selected a random sample of 1980 Ipsos Panel members to participate. Those eligible were ages 18+, and proficient with English or Spanish languages. Sixty-four percent (64.0%; n = 1267) of the recruited respondents completed the survey. The median survey completion time was 17 min. Upon completion, qualified respondents received a standard incentive payment from Ipsos (the cash-equivalent of $1) and entered into a sweepstakes to win prizes of up to $500. All study protocols were reviewed and approved by the Social and Behavioral Research Institutional Review Board at Tufts University, Boston, MA.

### 2.2 Measures

We used items from standardized surveys when available but there were limited number of validated COVID-19 survey measures at the time this study was developed. When not available, we adapted items from existing surveys, as described below. Our dependent variable (vaccine intention) was assessed by the following: *“If a vaccine became available to prevent the Coronavirus, would you get it?”* with response options “yes”, “no,” or “don’t know.” To assess perceptions about vaccine safety and efficacy, we asked whether participants believed that: *“Most vaccines are very safe”* and *“Most vaccines are very effective”* with response options on a 5-point Likert scale (strongly agree to strongly disagree), with higher scores indicating greater agreement. Based on items from the Vaccine Confidence Index, (Betsch et al., 2018) we assessed attitudes toward individual responsibility about vaccination by inquiring about level of agreement with three statements: *“I have a responsibility to get vaccinated because I can protect others with a weaker immune system,” “Vaccination is something everyone should do to protect others in the community,”* and *“When everyone else is vaccinated, I don't need to be vaccinated.”* Responses were on a 5-point Likert scale (strongly agree to strongly disagree), with higher scores indicating greater agreement. For beliefs about public authorities and vaccination, we adapted items from the Vaccine Confidence Index (Betsch et al., 2018) to assess respondents’ level of agreement with two statements: *“Public authorities decide about which vaccines to recommend based on the best interest of the community”* and *“Public authorities should be able to mandate that everyone be vaccinated”* with responses on a 5-point Likert scale (strongly agree to strongly disagree), with higher scores indicating greater agreement. In addition, we asked about personal and family history of COVID-19 infection, as well as questions regarding underlying health conditions believed to be associated with risk of severe COVID-19 consequences at the time of this survey (Centers for Disease Control and Prevention, 2020) (see Appendix 1). For each individual, we calculated the total number of underlying health conditions associated with elevated risk of severe COVID-19, and created three groups (0, 1–2, 3 + conditions) based on univariate distribution.

We drew socio-demographic measures, including age (continuous), gender (male/female), education (less than high school/high school/some college/bachelor’s degree or higher), race/ethnicity (non-Hispanic White/non-Hispanic Black/non-Hispanic Asian/ Hispanic/multiracial or other), employment status (working/not working), marital status (married or living with partner/other), religious affiliation (Catholic/ Protestant/other religion/unaffiliated), health insurance (employer provided/governmental insurance or marketplace/no insurance/other insurance), and party affiliation (Democrat/Republican/other) from the Ipsos Core Profile Survey. We calculated a ratio comparing household income to the household-size adjusted 2020 Federal Poverty Line (Office of the Assistant Secretary for Planning and Evaluation, 2020).

### 2.3 Statistical analyses

We excluded 48 respondents with missing data for the dependent variable (intention to vaccinate) and key independent variables (perceived vaccine safety and efficacy, individual/societal responsibility to vaccinate, and trust in the role of public authorities), yielding a final analytic sample of 1219. Since missing data only accounted for 3.8% (48/1219) of the sample, the parameter estimates were not likely to be biased enough to substantially change the results. We assessed measures of central tendency and distributions for continuous variables, as well as frequencies and proportions for categorical variables. We evaluated bivariate associations between vaccination intention, key independent variables, and potential confounders through Chi-square tests to select measures for consideration in final adjusted models. Variables that were associated with vaccination intention at a *p* < 0.10 level were considered for inclusion in multivariable models.

We found that two pairs of independent variables were highly correlated, so we created composite measures of the average Likert score of each pair. Specifically, we found that *“Most vaccines are very safe”* and *“Most vaccines are very effective”* had a high correlation (0.82). As a result, we combined them into a new variable (“*Most vaccines are safe/effective”),* which represents their numeric average. We also found that the correlation coefficient between the two items *“I have a responsibility to get vaccinated because I can protect others with a weaker immune system” and “vaccination is something everyone should do to protect others in the community”* was 0.89, so these items were combined into a new variable (“I/Everyone should vaccinate”*),* which is the numeric average of their Likert scales.

We constructed multinomial logistic regression models to identify associations between our key independent measures and vaccination intentions, adjusted for covariates. Given highly politicized views of the pandemic response, we also estimated multinomial logistic regression models to identify associations between our key independent measures and vaccination intentions stratified by political party affiliation and adjusted for remaining covariates. Variance inflation factors for covariates were accepted if they were less than <4.0. We present regression results as relative-risk ratios (RRRs), as advised by the technical notes in Stata (StataCorp., 2021) and similar practice. (Reiter et al., 2020) The interpretation is that RRRs are equal to the ratio of odds ratios in binary logistic regressions. All analyses applied sample weights to be more representative of the U.S. population. More information of sample weighting and survey design is available elsewhere. (Stopka et al., 2020) All analyses were conducted in Stata v.16.

## 3 Results

### 3.1 Sample characteristics

The analytic sample (n = 1219) was 52.1% female, with a mean age of 48.1 years (linearized standard error = 0.6 years). Approximately two-thirds (63.4%) were non-Hispanic White. About a third (32.3%) had a household income <$50,000, and about a third of respondents (33.5%) had attained a bachelor’s degree or more education. At the time of initial panel entry (78% in 2019), 65.6% were employed. Among those who did not refuse or have missing data on religion, nearly three-quarters (73.3%) reported being affiliated with a religious denomination, with a plurality (34%) reporting that they were Protestant. Approximately one-third of the sample (35.7%) were Democrats, 28.5% were Republicans, and 35.8% were Independents or from another party. Two-thirds (67.6%) reported having one or more underlying health conditions that placed them at higher risk for severe disease. Relatively few respondents believed that they had Coronavirus (5.9%) or reported that a family member had been diagnosed with the infection (4.1%). See Table 1.

**Table 1:** Characteristics of survey sample, United States, May 29-June 10, 2020 (n = 1219).^±.^

Characteristic	N (weighted %)
**Gender**	
Men	610 (47.9%)
Women	609 (52.1%)
**Age**	
18–29	123 (20.3%)
30–44	265 (25.3%)
45–59	334 (25.2%)
60+	497 (29.2%)
**Race/Ethnicity**	
NH White	862 (63.4%)
NH Black	111 (11.7%)
NH Asian	47 (5.9%)
Hispanic	146 (16.7%)
Multiracial or Other	53 (2.3%)
**Income**	
Less than $19,999	89 (9.5%)
$20,000 to $49,999	248 (22.8%)
$50,000 to $84,999	289 (25.0%)
$85,000 to $149,999	322 (23.4%)
$150,000 or more	271 (19.3%)
**Education**	
Less than high school	93 (10.6%)
High school	357 (27.9%)
Some college	322 (28.0%)
Bachelor's degree or higher	447 (33.5%)
**Employment Status**	
Working	776 (65.6%)
Not working	443 (34.4%)
**Marital Status**	
Married/living with partner	802 (59.9%)
Other	417 (40.1%)
**Religion**	
Catholic	306 (25.2%)
Protestant	437 (34.0%);
Other religion	167 (14.1%)
Unaffiliated	291 (25.1%)
Missing/refused	18 (1.6%)
**Health Insurance**	
Employer provided	624 (50.3%)
Governmental insurance or marketplace	407 (31.8%)
No Insurance	65 (7.1%)
Other insurance	35 (2.7%)
Missing/refused	88 (8.0%)
**Believe Infected by Coronavirus**	
Yes	70 (5.9%)
Don't know/refused	170 (14.1%)
**Family Member Infected with Coronavirus**	
Yes	48 (4.1%)
Don't know/refused	32 (3.4%)
**Party Affiliation**	
Democrat	411 (35.7%)
Republican	383 (28.5%)
Other	425 (35.8%)
Underlining Health Conditions*	
0 Condition	357 (32.4%)
1–2 Conditions	660 (53.9%)
3 + Conditions	202 (13.6%)

±Sample weights applied to be representative of U.S. population.

Percentages may not add up to 100% due to rounding.

*For full list, see Appendix 1.

### 3.2 Vaccine attitudes and intention to vaccinate

**Fig. 1:**
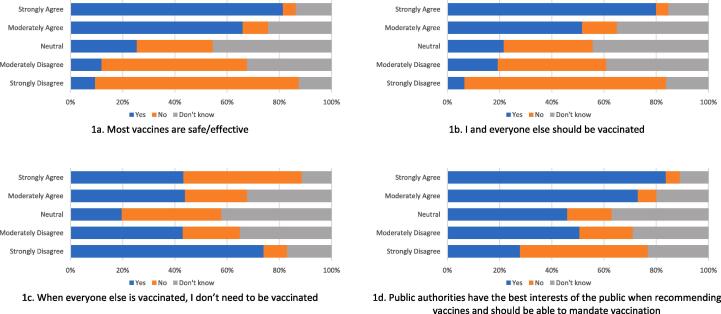
Vaccine attitudes and beliefs, stratified by intent to vaccinate, United States, May 29-June 10, 2020 (n = 1219). ± Blue = yes, intend to be vaccinated; orange = no do not intend to be vaccinated; gray = don’t know/unsure about vaccination. ± Sample weights applied to be representative of U.S. population. (For interpretation of the references to color in this figure legend, the reader is referred to the web version of this article.)

A total of 17.7% said that they would not get vaccinated and a quarter (24.2%) were unsure. Those who strongly disagreed with statements that most vaccines are very safe and/or effective were more likely to say they would not get the vaccine, compared with those who strongly believed most vaccines are very safe/effective (78.1% vs. 5.0%; Fig. 1a). Those who strongly disagreed with statements that they themselves and everyone else in society has a responsibility to be vaccinated were more likely to indicate they would not get a COVID-19 vaccine, compared with those who strongly agreed with these statements (77.2% vs 4.8%, Fig. 1b). When asked about not needing to be vaccinated if everyone else were vaccinated, results were mixed (Fig. 1c). Those who strongly opposed this statement were most likely to report an intention to vaccinate (74%), while those who strongly agreed were most likely to indicate they would *not* get the vaccine (45.3%). Those who were neutral about this statement were the most likely to report that they were unsure about vaccination (42.4%). Regarding trust of public authorities, half (49%) of those who strongly disagreed that public authorities decide about which vaccines to recommend based on the best interests of the community and that public authorities should be able to mandate vaccination reported that they did not intend to be vaccinated (Fig. 1d, orange bar).

### 3.3 Bivariate associations of factors with intention to be vaccinated

Gender, age, income, education, religious affiliation, insurance status, and political party affiliation were significantly associated with vaccine attitudes (Table 2). Although there was variation in vaccine intentions by race/ethnicity, with 15.8% of non-Hispanic White, 28.8% of non-Hispanic Black, 19% of Hispanics, and 15.3% of Asians reported that they would not get vaccinated, these differences were not statistically significant (p = 0.074).

**Table 2:** Sample characteristics by COVID-19 vaccination intentions, United States, May 29-June 10, 2020 (n = 1219).^±^

	Intend to vaccinate	Do not intend to vaccinate	Unsure about intentions	p value
	728 (58.2%)	197 (17.7%)	294 (24.2%)	
**Gender**				0.038
Men	386 (62.4%)	96 (16.3%)	128 (21.3%)	
Women	342 (54.3%)	101 (18.9%)	166 (26.8%)	
**Age**				0.005
18–29	82 (65.3%)	21 (16.9%)	20 (17.8%)	
30–44	141 (50.8%)	48 (19.7%)	76 (29.5%)	
45–59	183 (53.7%)	71 (22.6%)	80 (23.8%)	
60+	322 (63.5%)	57 (12.2%)	118 (24.2%)	
**Race/Ethnicity**				0.074
NH White	526 (59.2%)	128 (15.8%)	208 (24.9%)	
NH Black	54 (49.5%)	28 (28.8%)	29 (21.6%)	
NH Asian	28 (58.4%)	7 (15.3%)	12 (26.2%)	
Hispanic	88 (60.4%)	28 (19.0%)	30 (20.6%)	
Multiracial or Other	32 (56.1%)	6 (7.4%)	15 (36.5%)	
**Income**				<0.001
Less than $19,999	39 (42.7%)	20 (25.9%)	30 (31.4%)	
$20,000 to $49,999	122 (48.9%)	52 (20.9%)	74 (30.3%)	
$50,000 to $84,999	162 (56.5%)	59 (20.4%)	68 (23.1%)	
$85,000 to $149,999	206 (62.9%)	46 (15.8%)	70 (21.3%)	
$150,000 or more	199 (73.2%)	20 (8.6%)	52 (18.2%)	
**Education**				<0.001
Less than high school	48 (51.5%)	24 (25.8%)	21 (22.7%)	
High school	153 (41.1%)	83 (25.0%)	121 (33.8%)	
Some college	194 (59.3%)	57 (18.8%)	71 (21.9%)	
Bachelor's degree or higher	333 (73.6%)	33 (8.0%)	81 (18.4%)	
**Employment**				0.164
Working	462 (58.9%)	135 (18.7%)	179 (22.4%)	
Not working	266 (56.8%)	62 (15.7%)	115 (27.5%)	
**Marital Status**				0.335
Married/living with partner	496 (60.1%)	123 (16.6%)	183 (23.3%)	
Other	232 (55.3%)	74 (19.3%)	111 (25.4%)	
**Religion**				0.002
Catholic	203 (65.5%)	33 (11.8%)	70 (22.7%)	
Protestant	230 (51.2%)	86 (21.8%)	121 (27.1%)	
Other religion	98 (53.8%)	32 (22.4%)	37 (23.8%)	
Unaffiliated	189 (64.0%)	43 (15.5%)	59 (20.5%)	
Missing/refused	8 (38.1%)	3 (15.9%)	7 (46.0%)	
**Health Insurance**				0.01
Employer Provided	387 (61.3%)	87 (14.7%)	150 (24.1%)	
Governmental/marketplace	246 (56.7%)	59 (17.9%)	102 (25.4%)	
No Insurance	26 (41.4%)	25 (35.8%)	14 (22.8%)	
Other insurance	21 (62.9%)	8 (20.6%)	6 (16.5%)	
Missing/refused	48 (57.9%)	18 (18.3%)	22 (23.9%)	
**Believe Infected by Coronavirus**				0.165
No	575 (57.0%)	162 (18.2%)	242 (24.7%)	
Yes	41 (58.0%)	14 (24.1%)	15 (18.0%)	
Don't know/refused	112 (64.7%)	21 (11.9%)	37 (23.4%)	
**Family Member Infected by Coronavirus**				0.635
No	687 (58.9%)	182 (17.4%)	270 (23.7%)	
Yes	24 (50.5%)	9 (22.7%)	15 (26.8%)	
Don't know/refused	17 (48.8%)	6 (18.0%)	9 (33.2%)	
**Party Affiliation**				<0.001
Democrat	300 (71.0%)	33 (11.1%)	78 (17.9%)	
Republican	191 (46.7%)	93 (26.8%)	99 (26.5%)	
Other	237 (54.5%)	71 (17.0%)	117 (28.5%)	
Underlining Health Conditions*				0.313
0 Condition	230 (62.6%)	57 (17.0%)	70 (20.4%)	
1–2 Conditions	378 (55.9%)	107 (18.0%)	175 (26.2%)	
3 + Conditions	120 (56.7%)	33 (18.0%)	49 (25.3%)	

± Sample weights applied to be representative of U.S. population.

*For full list, see Appendix 1.

Percentages may not add up to 100% due to rounding.

### 3.4 Multivariable modeling

In multinomial logistic regression models adjusted for key covariates (Table 3), those who believe that most vaccines are safe and/or effective were less likely to say they do not intend to be vaccinated, compared with those who disagreed with this statement (relative risk ratio (RRR) = 0.45, 95% confidence interval (CI) = 0.31, 0.66). Similarly, people who agreed that they (themselves) and everyone else should be vaccinated were less likely to report that they would not be vaccinated versus being vaccinated (RRR = 0.39, 95% CI = 0.30, 0.52) or being unsure about vaccination intention (RRR = 0.60, 95% CI = 0.47, 0.75). Conversely, those who agreed that if everyone else were vaccinated they would not need to be vaccinated were significantly more likely to report unwillingness to be vaccinated compared with willingness to get vaccinated (RRR: = 1.36, 95% CI: = 1.04, 1.78) or being unsure about vaccination intentions (RRR = 1.26, 95% CI = 1.05, 1.53). Those who agreed with the statement that public authorities should be able to mandate vaccinations were also less likely to say they would not get the vaccine than would get the vaccine (RRR = 0.75, 95% CI = 0.58, 0.98) or than being unsure about whether to vaccinate (RRR = 0.82, 95% CI = 0.71, 0.95).

**Table 3:** Associations with vaccination intentions, United States, May 29-June 10, 2020 (n = 1219).±

	RRR [95%CI]
**Intend not to vaccinate compared to intended to vaccinate**	
Most vaccines are safe and/or effective	0.45**
	[0.31, 0.66]
I/Everyone should get the vaccine	0.39**
	[0.30, 0.52]
If everyone else is vaccinated, I don't need to	1.36*
	[1.04, 1.78]
Public authorities hold the best interests of public	0.82
	[0.62, 1.07]
Public authorities should be able to mandate vaccination	0.75*
	[0.58, 0.98]
**Unsure compared to intend to vaccinate**	
Most vaccines are safe and/or effective	0.59**
	[0.46, 0.77]
I/Everyone should get the vaccine	0.60**
	[0.47, 0.75]
If everyone else is vaccinated, I don't need to	1.26*
	[1.05, 1.53]
Public authorities hold the best interests of public	0.99
	[0.84, 1.17]
Public authorities should be able to mandate vaccination	0.82*
	[0.71, 0.95]

RRR: Relative Risk Ratio; CI: confidence interval * p < 0.05; ** p < 0.01 ± Sample weights applied to be representative of U.S. population.

Covariates in both models include: gender (men, women); age (continuous); race/ethnic status (non-Hispanic White, non-Hispanic Black, non-Hispanic Asian, Hispanic, multi-racial or other); household income as a percentage of Federal Poverty Level^27^; education (less than high school, high school, some college, bachelor's degree or higher); religion (Catholic, Protestant, other religion, unaffiliated, missing/refused); health insurance (employer provided, governmental insurance or marketplace, no insuranc

### 3.5 Effect modification by political party affiliation

Political party affiliation was strongly associated with vaccine intentions in bivariate analyses. Due to this partisan difference, as well as recent findings from other polls and studies, (Bokemper et al., 2021) we repeated the primary multivariable analyses stratified by party affiliation. Overall trends in stratified models were generally consistent with the primary analysis, and we did not observe substantial evidence for effect modification by party affiliation on the relative scale (Appendix 2). One exception was for the relationship between agreement with the statement “public authorities should be able to mandate that everybody be vaccinated” and vaccine intentions. Those who agreed with this statement were less likely to report that they would not get a vaccine (versus intending to get vaccinated) among Democrats (RRR = 0.52, 95% CI = 0.34, 0.81) and Republicans (RRR = 0.53, 95% CI = 0.29, 0.99), but not among those from other political parties (RRR = 1.39, 95% CI = 0.92, 2.09).

## 4 Discussion

In a national survey of U.S. adults administered between May-June, nearly one in five (17.7%) reported that they would not get a COVID-19 vaccine when it became available and one in four respondents remained unsure. Those who reported that they would not be vaccinated were more likely to have concerns about vaccine safety and efficacy, to believe that there is not an individual or societal responsibility to be vaccinated, and to oppose vaccine mandates compared to those who reported that they would get a vaccine. Moreover, those who said they would not be vaccinated were more likely to believe that they would not need a vaccine if everyone else were vaccinated. These findings can help to inform interventions designed to address the concerns and attitudes of those Americans least receptive to taking the vaccine and could help to inform public health messaging as vaccination continues to be rolled out in 2021.

Our findings regarding the percentage of the U.S. population reporting that they would not be vaccinated are in line with U.S. polls conducted in the spring of 2021, which reported that around 13–15% of adults would ‘definitely not’ be vaccinated. (Huetteman, 2021) Whereas there are available U.S. polls that provide data on socio-demographic characteristics of those who intend to be vaccinated, there are a limited number of published studies in the U.S. that have reported specifically on cognitive, attitudinal, and political factors associated with vaccine refusal. (Reiter et al., 2020, Fisher et al., 2020, Head et al., 2020, Malik et al., 2020) Several U.S. studies have observed that concerns about vaccine safety and efficacy are associated with lower likelihood of being vaccinated (Reiter et al., 2020, Head et al., 2020, Guidry et al., 2021) Altruism was associated with higher intention to be vaccinated in two other recent studies. (Head et al., 2020, Edwards et al., 2021) We believe that our study adds new insights into the issue of vaccination, in that we include cognitive, attitudinal *and* normative factors in our analysis.

Research to date suggests that acceptance of COVID-19 vaccination varies substantially within and among countries. (Sallam, 2021) Several international studies have found that some psychological constructs, such as trust in public authorities and altruism, may underlie vaccine intentions across populations. (Edwards et al., 2021, Rozbroj et al., 2019, Murphy et al., 2021) While it is important to understand the unique socio-cultural and political contexts in which country-based vaccination efforts are taking place, results from our study may be instructive for domestic and international COVID-19 vaccination programs. For example, perceptions regarding vaccine mandates may differ by political orientation; while mandates may be acceptable among those with more liberal leanings, this may not be the case for those who are more identified with conservative beliefs. In the context of COVID-19, there is evidence to suggest that political ideologies play an even bigger role in attitudes toward vaccine mandates than for other vaccine-preventable illnesses. (Haeder, 2021)

Several limitations affect the interpretation of our results. First, we cannot draw causal inferences given the cross-sectional design. Second, the actual characteristics of COVID-19 vaccines (e.g., efficacy, number of required doses) were not known at the time the survey was conducted. We also recognize that attitudes toward vaccination may shift over time. (Fridman et al., 2021) Regardless, the percentage of the U.S. population that say that they will decline vaccination has remained relatively unchanged over the past year. Finally, like many national surveys, we may not have had sufficient statistical power to examine differences among race/ethnicity groups. Reassuringly, the weighted percentages of our sample by gender, age, race/ethnicity, marital status, and income were comparable to those of the 2019 U.S. Census Bureau’s Current Population Survey.

Despite these limitations, the study has important strengths, including the large, national sample designed to be representative of the U.S. population and the broad array of information collected about characteristics that may influence vaccination intentions. As such, these findings provide timely information that can help to guide policy and practice. Specifically, with our result that nearly 18% of the population reported that they would not get the COVID-19 vaccine, it is critical to address concerns about the safety and efficacy of vaccines, attitudes toward vaccination, and trust in public authorities.

## 5 Implications

Our findings may be helpful in identifying groups who are reluctant to be vaccinated or who face barriers to getting vaccinated. While those who reject vaccination outright constitute a fraction of the U.S. population, their resistance could result in outbreaks of disease and exacerbate concerns about viral mutations. It will likely require less effort to ‘tip the balance’ in favor of vaccination among those who are unsure, (Attwell et al., 2021) particularly as more recent studies point to lack of access as being an impediment to achieving high uptake among those who are not wholly resistant. (Pham and Alam, 2021) Our observations suggest that it may be particularly important to reach out to those with lower levels of income, for example, as they are disproportionately impacted by the pandemic. (Finch and Hernández Finch, 2020) Providing information about availability of free vaccines in accessible locations may help to reduce some barriers, such as cost and transportation issues. This is particularly important as data show that although communities of color face a significantly higher risk of COVID-19 infections and deaths (Centers for Disease Control and Prevention, 2020) and no longer represent a substantial portion of those who are reluctant to take the vaccine, non-Hispanic White populations are more likely to have already been vaccinated. (McLeroy et al., 1988)

The socio-ecological model can provide a useful framework for interventions to promote vaccination. (Janz and Becker, 1984) At the individual level, our findings suggest that educational messages should stress the rigorous and ethical process through which vaccines were developed and tested and emphasize that safety and efficacy were not sacrificed by the expedited timeframe for development and testing. Such messaging is consistent with the Health Belief Model, (Rosenthal et al., 2011) which stresses the importance of addressing perceived benefits and barriers to vaccination. In addition, our finding that those who did not intend to be vaccinated were more likely to endorse the idea that they would not need to be vaccinated if others were, suggests a lack of understanding about the importance of herd immunity. Stressing the importance of this, both for protection of others and to limit opportunities for viral mutation may be key. At the interpersonal and community levels, development of interventions to create injunctive norms about vaccination could be particularly helpful in increasing vaccination rates. For example, messaging that appeals to the desire to protect one’s family members (e.g., “do this for grandmother”) and a sense of collectivism (e.g., “we are all in this together”) could be bolstered by messaging that promotes vaccines as normative (e.g., “we all have to do our part”).

Our findings suggest that those opposed to vaccination are less trusting of public authorities. Across a variety of (non-COVID) vaccines, having a healthcare provider recommendation has been shown to be the most influential factor in individual decision-making. (Bokemper et al., 2021, Jacobson et al., 2020) Therefore, ensuring that vaccine recommendations are consistently made by healthcare providers is vital. Prior studies on other vaccine types find that offering vaccines at all visits (“points of care”) using a presumptive approach (i.e., assumes that a patient will be vaccinated) is more effective than inquiring about vaccine hesitancy, (Institute of Medicine (US) Committee on Review of Priorities in the National Vaccine Plan, 2010) although an informed decision-making approach, which stresses providing information for an individual to make their own decision, has also been recommended. (de Beaumont Foundation, 2021) Given partisan attitudes toward control of the pandemic across the U.S., it may be important for political leaders of both parties to convey the importance of vaccination – or, for politicians to take a step back from debates about vaccines and allow public health experts and other trusted community leaders (e.g., faith leaders) to deliver the message. (Schoch-Spana et al., 2020, Latkin et al., 2021) Dissemination of information through media outlets that attract those with conservative political ideologies may also hold promise.

## Funding

This research was supported by the Tufts University Office of the Vice Provost for Research (Jennifer Allen, Thomas Stopka). Laura Corlin was supported by NICHD K12HD092535 and by the Tufts/TMC COVID-19 Rapid Response Seed Funding Program. Thomas Stopka was funded by the Tufts/TMC COVID-19 Rapid Response Seed Funding Program.

## Declaration of Competing Interest

The authors declare that they have no known competing financial interests or personal relationships that could have appeared to influence the work reported in this paper.
